# Effectiveness of internet-based cognitive behavioural therapy for binge eating disorder

**DOI:** 10.1192/j.eurpsy.2021.923

**Published:** 2021-08-13

**Authors:** E. Jensen, J. Linnet, T. Holmberg, K. Tarp, J. Nielsen

**Affiliations:** Research Unit For Telepsychiatry And Emental Health, Centre for Telepsychiatry, Odense C, Denmark

**Keywords:** binge eating disorder, eMental health, effectiveness, Internet-based treatment

## Abstract

**Introduction:**

Binge eating disorder (BED) is the most prevalent specific eating disorder. It is characterized by recurrent episodes of binge eating and is associated with feelings of shame and a lack of control. Internet-based treatments are gaining increasing attention as a way to reach more patients with evidence based treatments In 2020 we conducted a preliminary analysis on the effectiveness of an internet-based cognitive behavioural therapy treatment project (Jensen ES, Linnet, J, Holmberg TT, Tarp K, Nielsen JH, Lichtenstein MB. Effectiveness of internet-based guided self-help for binge-eating disorder and characteristics of completers versus noncompleters. Int J Eat Disord. 2020;1-6. https://doi.org/10.1002/eat.23384).

**Objectives:**

This study aims to update the analyses on treatment effect with the patients who have completed treatment in the year following the last data extraction.

**Methods:**

The iBED treatment project is a 10-session psychologist guided internet-based self-help program based on cognitive behavioural therapy. When applying for treatment and upon completion patients respond to a survey containing, among other scales, the eating disorder examination-questionnaire (EDE-Q), binge eating disorder-questionnaire (BED-Q) and various sociodemographic questions. Data will be extracted from the treatment project in anonymized form for analyses.

**Results:**

The preliminary analyses were conducted on 36 completers. These showed large standardized effect sizes on both the EDE-Q subscales (Cohens d ranging from .88-1.65) and on the BED-Q (d = 1.38). The updated effectiveness analyses will be presented at the conference. We expect approximately 70-80 patients to have completed treatment at this time.

**Conclusions:**

Results will be discussed and presented at the conference.

